# An Update on *Clostridioides difficile* Binary Toxin

**DOI:** 10.3390/toxins14050305

**Published:** 2022-04-27

**Authors:** Adrián Martínez-Meléndez, Flora Cruz-López, Rayo Morfin-Otero, Héctor J. Maldonado-Garza, Elvira Garza-González

**Affiliations:** 1 Subdirección Académica de Químico Farmacéutico Biólogo, Facultad de Ciencias Químicas, Universidad Autónoma de Nuevo León, Pedro de Alba S/N, Cd Universitaria, San Nicolás de los Garza 66450, Nuevo Leon, Mexico; adrian.mtz.fcq@gmail.com (A.M.-M.); flora.cruz@live.com (F.C.-L.); 2 Instituto de Patología Infecciosa y Experimental “Dr. Francisco Ruiz Sánchez”, Centro Universitario de Ciencias de la Salud, Universidad de Guadalajara, Calle Hospital 308, Colonia el Retiro, Guadalajara 44280, Jalisco, Mexico; rayomorfin@gmail.com; 3 Servicio de Gastroenterología, Facultad de Medicina/Hospital Universitario “Dr. José Eleuterio González”, Universidad Autónoma de Nuevo León, Av. Francisco I. Madero Pte. S/N y Av. José E. González, Col. Mitras Centro, Monterrey 64460, Nuevo Leon, Mexico; hectormaldonadog@yahoo.com; 4 Departamento de Bioquímica y Medicina Molecular, Facultad de Medicina y Hospital Universitario “Dr. José Eleuterio González”, Universidad Autónoma de Nuevo León, Av. Francisco I. Madero Pte. S/N y Av. José E. González, Col. Mitras Centro, Monterrey 64460, Nuevo Leon, Mexico

**Keywords:** *Clostridioides difficile*, binary toxin, epidemiology, hypervirulent strains

## Abstract

Infection with *Clostridioides difficile* (CDI), a common healthcare-associated infection, includes symptoms ranging from mild diarrhea to severe cases of pseudomembranous colitis. Toxin A (TcdA) and toxin B (TcdB) cause cytotoxicity and cellular detachment from intestinal epithelium and are responsible for CDI symptomatology. Approximately 20% of *C. difficile* strains produce a binary toxin (CDT) encoded by the *tcdA* and *tcdB* genes, which is thought to enhance TcdA and TcdB toxicity; however, the role of CDT in CDI remains controversial. Here, we focused on describing the main features of CDT and its impact on the host, clinical relevance, epidemiology, and potential therapeutic approaches.

## 1. Introduction

*Clostridioides difficile* is a Gram-negative, anaerobic, spore-forming, and toxin-producing bacillus [1]. Infections with *C. difficile* (CDI) include symptoms ranging from mild diarrhea to severe cases of pseudomembranous colitis [2]. CDI is a frequently reported healthcare-associated infection in the USA, with 500,000 estimated cases and 29,000 deaths per year [3]. *C. difficile* colonizes the intestinal tract in humans and other mammals; in humans, up to 70% of infants and 5% of adults are colonized by this microorganism [4]. *C. difficile* spores are resistant to environmental desiccation and disinfectants and therefore persist on surfaces for years [1]. Spores are activated in the intestinal tract because of microbiota dysbiosis caused by consumption of antibiotics; other risk factors for CDI development are extended hospital stays, age > 65 years, immunosuppression, transplants, and cancer [1]. Two toxins produced by *C. difficile*, the Rho glycosylases toxin A (TcdA) and toxin B (TcdB), are responsible for CDI symptomatology; they cause cytotoxicity, cellular detachment from intestinal epithelium, and inflammation at the infection site [5]. TcdA and TcdB are encoded by the pathogenicity locus (PaLoc), which includes five genes: *tcdA* (toxin A), *tcdB* (toxin B), and three regulatory genes. Approximately 20% of *C. difficile* strains produce a binary toxin (CDT) encoded by the *cdtA* and *cdtB* genes. CDT is thought to enhance TcdA and TcdB toxicity and is related to more severe disease and higher sporulation rates [4,5].

A strain denominated BI/North American PFGE type 1 (NAP1)/027 [6] is positive for binary toxin and has been associated with an increased production of toxins A and B owing to mutations in the toxin regulatory gene *tcdC* [7]; additionally, the production of the binary toxin has been linked to more severe disease [8], but the role of CDT in CDI remains controversial. In the present study, we focused on describing the main features of CDT and its impact on the host and clinical relevance, epidemiology, and diagnostic and therapeutic approaches. We described recent findings in epidemiology and therapeutic approaches due to the increasing relevance of CDT in disease, mainly in the area of chaperone inhibitors.

## 2. *Clostridioides difficile* Binary Toxin (CDT)

**Figure 1:**
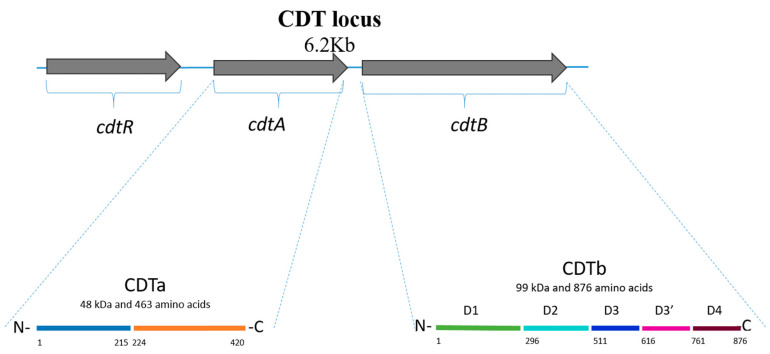
Representation of CDT locus and CDTa and CDTb components [11,17,18].

*C. difficile* binary toxin is an actin-ADP-ribosylating protein that belongs to a family of binary toxins produced by *C.* *botulinum* (C2 toxin), *C.* *perfringens* (iota toxin), *C.* *spiroforme* (toxin CST), *Bacillus anthracis* (edema and lethal toxins), and *B. cereus* (vegetative insecticidal proteins) [9]. The toxin is encoded by the *cdtA* and *cdtB* genes, located within a 6.2 kb region designated the “CDT locus” (CDTloc; Figure 1) [10,11]. In addition, CDTloc contains the *cdtR* gene, which encodes LytTR family response regulator [12]. CdtR is involved in the positive regulation of CDT production as well as TcdA and TcdB production; this regulation occurs at the transcriptional level, possibly via indirect regulation of TcdR, a positive regulator of PaLoc gene expression [13,14,15]. CdtR is activated by phosphorylation of Asp61 in RT027 strains; however, in RT078 strains, CdtR has demonstrated a lack of function due to polymorphisms in the promoter region, potentially suggesting a mechanism of evolution [16].

The mechanism of CDT secretion is currently unknown because CDT does not contain secretory signals and no genes are associated with its transport [11].

CDT comprises two regions, CDTa (48 kDa in size) and CDTb (99 kDa). CDTa is divided into two domains: the N-terminal part (residues 1 to 215), which interacts with CDTb, and the C-terminal part (residues 224 to 420), which catalyzes the ADP-ribosylation of actin [11,12,17]. CDTa comprises 463 amino acids and has a mass of ~53 kDa; the first 43 amino acids are cleaved by proteolysis, leaving a CDTa protein with a mass of ~48 kDa (Figure 1) [17]. CDTb comprises 876 amino acids and four conserved domains: D1 formed by 295 residues (at the N-terminus), D2 formed by amino acids 296 to 511, D3 formed by residues 512 to 615, and D4 formed by residues 761 to 876. These domains are involved in activation (D1, after proteolytic cleavage of a ~20 kDa fragment at the N-terminus), pore formation and membrane insertion (D2), oligomerization (D3), and receptor binding (D4). A fifth domain recently described is called D3′ (residues 616 to 744), and it is contained between D3 and D4; this domain is thought to encode for a galactose binding site [18].

**Figure 2:**
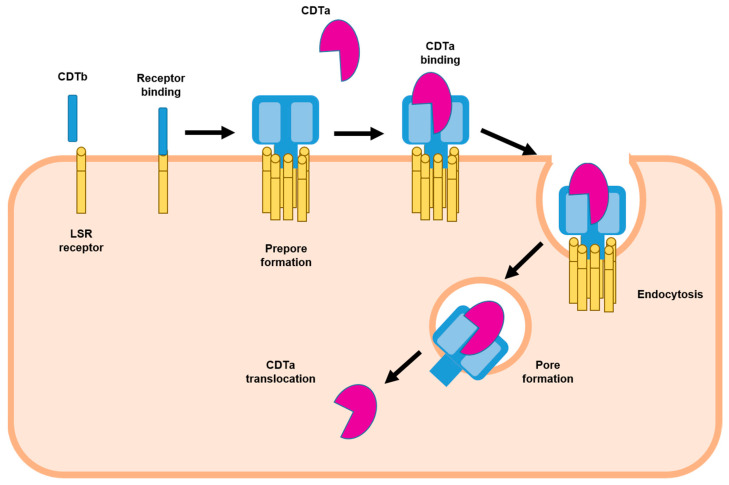
Representation of receptor binding and cell entry of CDT [11,17,18].

Refs. [11,12] CDTb also contains a signal sequence of 42 amino acids (Figure 1) [17]. The lipolysis-stimulated lipoprotein receptor (LSR) was identified as the host cell receptor for CDT [19]. CDTb induces the clustering and accumulation of the receptor into lipid rafts, and the N-terminus of CDTb serves as a binding site for CDTa (Figure 2) [10]. The local accumulation of CDTb monomers promotes oligomerization of CDTb on the cell surface, and the enzymatic component (CDTa) in turn binds to CDTb, thus triggering internalization of this complex into cells [12]. The low pH of endosomes probably induces the insertion of the binding component into the membrane and allows pore formation to deliver the toxin into the cytosol [17]; CDT inserts into the vesicle membrane to form a transmembrane β-barrel channel [20]. It has been described that translocation is dependent on host helper proteins such as Hsp90, FK506-binding protein 51, and peptidyl-prolyl cis/trans isomerase cyclophilin A [17]. Although proteolytic activation of the transport component is not essential for receptor binding and clustering into lipid rafts, it is required for oligomerization and subsequent intoxication of host cells [10].

## 3. In Vitro Effects of CDT

The main effects induced by CDT include cell rounding, inhibition of migration, and activation of leucocytes [12]. CDTa ADP-ribosylates G-actin at Arg177, which in turn inhibits actin polymerization [21]. ADP-ribosylated actin then acts as a capping protein, which inhibits polymerization of non-modified actin, eventually resulting in complete depolymerization of the actin cytoskeleton, thus causing changes in cell morphology and tight junctions [12].

CDTb causes cell rounding and damage in Vero and CaCo-2 cell monolayers, with loss of cell viability and epithelial integrity; the latter depends on the presence of the LSR, the specific cellular receptor of CDT [22]. However, when acting alone, CDTb does not induce cell rounding and is inhibited by enzymatically inactive CDTa or a pore blocker, suggesting that CDTb induces the production of pores in cytoplasmic membranes, thus contributing to cytotoxicity [22]. Furthermore, CDTb and the receptor-binding domain (RBD) of CDTb induces clustering of LSR into sub-compartments that contain marker proteins of lipid rafts; oligomerization occurs at the membrane and is enhanced by local accumulation of LSR-bound monomers into lipid rafts [23]. CDT induces microtubule redistribution and formation of protrusions at the surface of intestinal epithelial cells; this occurs together with ADP ribosylation of actin and depolymerization of microfilaments [24]. Electron microscopical studies have shown that protrusions increase the adherence of *C. difficile* by five-fold at the cell surface of epithelial cells under anaerobic conditions; thus, this mechanism enhances colonization of the pathogen [25]. However, it has been demonstrated that TcdA and TcdB negative, but binary toxin positive strains (A^−^B^−^C^+^) are non-toxigenic in vitro. In a study by Kuehne et al., a series of toxin A and B mutants in which the binary toxin genes were still functional were assayed in vitro and in vivo. The strains expressing toxin B and CDT (A^−^B^+^C^+^) as well as those expressing toxin A and CDT (A^+^B^−^C^+^) showed cytotoxicity in vitro; moreover, in a golden Syrian hamster model, all the hamsters (8 of 8) developed terminal CDI. However, when evaluating the A^−^B^−^C^+^ mutants, three of nine animals succumbed to disease with no typical symptoms of CDI. Some hemorrhage and inflammation were observed in their small intestines, suggesting that *C. difficile* caused infection in the small intestine [26]. Furthermore, using a mouse infection model (in which animals rarely progress to severe disease or death), less intestinal damage was detected in animals infected with an A^−^B^−^CDT^+^ mutant than in wild-type strain-infected mice (*p* = 0.0022). Additionally, histopathological scoring of tissues from CDT^−^ strain-infected mice were similar to those found in wild-type strain-infected mice [27]. Additionally, it has been demonstrated that *cdtR* mutants produced less TcdA and TcdB than the wild type and when mutants were complemented, high levels of toxins A and B were detected, showing high cytotoxicity in vitro; also, the relative transcription of toxin A, B and CDT, as well as TcdR were significantly decreased in mutants compared to the wild type strain [14].

Little is known about CDT and its role in immune response; however, CDT may enhance the disruption of the host’s protective mechanisms stimulated by *C. difficile* toxins A and B [28]. It has been shown that CDT enhances the virulence of RT027 strains in animal models by inducing pathogenic host inflammation, resulting in eosinophil apoptosis in the colon and blood [29]. Furthermore, CDT activates NFκB and induces inflammatory interleukin (IL)-1β production by TLR2 signaling [29]. The subunit CDTb provokes mucosal-associated invariant T (MAIT) cell activation and degranulation of the lytic granule components. MAIT cell responses depend on IL-18 and the major histocompatibility complex (MHC) class I-related protein (MR1) signaling pathway. Additionally, CDT-stimulated monocytes seem to be involved in MR1-dependent activation of MAIT cells. Furthermore, it is suggested that MAIT cell cytotoxicity contributes to diminution of toxemia and the immunopathology of the disease [30].

## 4. CDT-Producing Ribotypes

*C. difficile* is a diverse and heterogeneous species, and CDI exhibits a changing epidemiology. CDT is produced by diverse PCR ribotypes including those considered as hypervirulent and epidemic, such as PCR ribotypes 027 and 078 [11]. Strains producing only CDT have been isolated from symptomatic patients, adding evidence for CDT as a contributor to the pathogenesis [31].

A^+^B^+^CDT^+^ ribotypes include PCR ribotype 027 (RT027) and PCR ribotype 078 (RT078). The prevalence of strain RT027 (Table 1) has increased since 2002 after the first reports in Canadian Hospitals, together with an increase in mortality and morbidity [32]. In Europe, this strain was reported in the Netherlands [33], and an association between the use of fluoroquinolones and CDI was described for the first time. RT027 is considered as “hypervirulent” owing to increased production of TcdA and TcdB, together with the production of binary toxin [8], which is associated with a higher production of toxin in vitro [34]. Furthermore, increased production of toxins has been linked to severe disease, 30-day all-cause mortality [35], and recurrent episodes [36]. In the Netherlands, the CDT^+^ RT078 (Table 1) [37] was reported in patients younger than those infected with RT027, and it was mainly community-associated (CA-CDI); moreover, rates of severe diarrhea and mortality induced by the CDT^+^ RT078 were similar to those induced by RT027 [37]. Furthermore, RT078 is associated with zoonotic transmission from pigs and cattle [38,39,40,41]. PCR ribotype 023 (Table 1) is another CDT^+^ strain with disease severity and attributable mortality comparable with that of hypervirulent strains (RT027 and RT078/126); furthermore, RT023 is associated with community-acquired cases [42]. RT244 is an RT027-related strain, associated with severe disease [43]; this strain is frequently reported in New Zealand, mainly in CA-CDI cases [44].

A^−^B^−^CDT^+^ *C. difficile* strains may contain additional antimicrobial resistance determinants that contribute to enhanced survival and colonization [68]. However, regarding susceptibility, RT033 isolates (Table 1) have been reported to be susceptible to fidaxomicin, rifaximin, vancomycin, metronidazole, amoxicillin/clavulanate, and meropenem but resistant to tetracycline, moxifloxacin, erythromycin, and clindamycin [68]. Phenotypic assays performed in a collection of 148 strains of 10 different ribotypes (033, 238, 239, 288, 585, 586, QX143, QX444, QX521, and QX629) showed that A^−^B^−^CDT^+^ strains, except RT239, were non-motile. However, the flagellin and flagella cap genes were conserved. Furthermore, the strains produced deoxyribonuclease, esterase, and mucinase; however, they were not found to be pathogenic in an animal model [69].

## 5. Clinical Relevance

CDT is considered a virulence factor that contributes to the severity of CDI, mainly in patients infected with hypervirulent strains; it has been shown that *C. difficile* strains, especially RT027 strains, express CDT in vitro. However, there is limited evidence for the role of CDT in the pathogenesis of CDI [70]. One of the initial studies regarding clinical impact of CDT was reported by Barbut et al. in a retrospective case–control study to identify clinical features and risk factors of CDI attributable to CDT^+^ strains. Most of the cases were community-acquired (65.4%). Diarrhea was associated with abdominal pain (*p* = 0.07) and with liquid stools (*p* = 0.14) [8]. Moreover, the presence of CDT has been described as an independent predictor of recurrent CDI, and binary toxin producers may require long antibiotic regimens [71]. Similarly, Stewart et al. found an association between the presence of a binary toxin gene with at least one recurrent episode of CDI (*p* = 0.03); furthermore, it predicted the need for hospital admission for a primary episode of CDI and the first recurrence [72]. Furthermore, Bacci et al. showed that patients infected with binary toxin-producing strains had higher case fatality rates than patients infected with other strains [73].

Recently, López-Cárdenas et al. studied the association between the binary toxin and the appearance of severe disease, complications, or recurrence; patients infected with CDT^+^ strains showed higher frequencies of severe disease than patients with CDT^−^ strains (39.2% vs. 21.2%, *p* = 0.005) and higher rates of complications and recurrence than patients with CDT^−^ strains (21.6% vs. 10.9%, *p* = 0.037 and 14.9% vs. 5.8%, *p* = 0.029; respectively). In total, 45.5% of CDT^+^/TcdB^+^ cases presented severe disease compared with 18.6% in the CDT^−^/TcdB^−^ group (*p* = 0.018), and the TcdB^+^/CDT^+^ group had significantly more complicated cases (33.3% vs. 10.3%, *p* = 0.013) and recurrences (24.2% vs. 5.2%, *p* = 0.031) [74], indicating that infection with TcdB^+^/CDT^+^ strains had a greater impact on prognosis. Regarding mortality, it has been reported that patients infected with a CDT^+^ strain were nearly eight times more likely to die than patients infected with CDT^−^ strains [75]. In addition, Goldenberg et al. showed that 28% of the 207 *C. difficile* isolates analyzed in a 2-year period possessed binary toxin genes. The white cell count and 30-day all-cause mortality rate were significantly higher in the CDT^+^ group [76].

However, despite the latter findings and associations of infection with CDT^+^ strains and severity or complications, there is no convincing epidemiological evidence that binary toxin is a marker of severe disease or complications [77,78]. A retrospective case–control study from Belgium compared clinical and epidemiological characteristics of 33 patients with binary toxin-positive CDI and 33 patients with binary toxin-negative CDI. The patients did not differ in disease recurrence, morbidity, or mortality, except for a higher peripheral leukocytosis in the binary toxin-positive group (16.30 10^9^/L vs. 11.65 10^9^/L; *p* = 0.02). Thus, the authors concluded that the presence of the binary toxin gene is not associated with poor outcome [79]. Additionally, Berry et al. analyzed clinical severity and outcome data of 1083 patients with CDI; the presence of binary toxin was associated with longer hospital stays and a higher risk of all-cause mortality (with a risk ratio of 1.68 [*p* < 0.001]). However, the presence of CDT did not predict the clinical severity of CDI [80]. Moreover, Reigadas et al. investigated the association between CDT^+^ isolates and outcome of 319 CDI cases in a non-027 ribotype setting; in total, 54 cases (16.9%) were caused by CDT^+^ strains, of which 90.7% belonged to ribotype 078/126. There were no differences in the rates of recurrent cases, treatment failure, overall mortality, or CDI-related mortality between infections caused by CDT^−^ and CDT^+^ strains. No association was found between the presence of CDT and poor outcome [81].

The described results depict the controversial status of CDT and its contribution to severity of diarrhea over the years; more clinical and fundamental research is needed to elucidate the level of virulence of CDT-producing strains.

## 6. Implications of CDT in Laboratory Diagnosis

The diagnosis of CDI is based on detection of *C. difficile* toxins in a stool sample. Cytotoxicity assays are the gold standard for detecting toxigenic *C. difficile* (toxins A and/or B) in the stool [82,83]. This technique has a sensitivity up to 100% and specificity up to 99%; however, it is labor-intensive and requires trained personnel; thus, it is not appropriate for routine diagnosis [82,83]. Due to this, detection of glutamate dehydrogenase (GDH) has been implemented, with a rapid turnaround time and a specificity of almost 100%. However, this test does not distinguish whether the strain is toxigenic (specificity of 59%); thus, GDH testing must be paired with a test that detects toxins [74,84,85,86]. Enzyme immunoassays (EIAs) detect toxins A and B; the sensitivity and specificity are variable (from 75–85% and specificities form 95–100%) depending on if the reaction is performed over a membrane or in a well-based EIAs [82,87]. Nucleic acid amplification testing (NAAT) is based on either a PCR method or isothermal amplification. These tests detect toxin genes (*tcdA* and *tcdB*), the *tcdC* gene, and/or CDT genes and identify the presence of toxigenic *C. difficile* in a single step [88,89,90]. NAAT testing shows sensitivity and specificity higher than 90%. However, due to its sensibility, NAAT test can detect toxigenic *C. difficile* in asymptomatic patients; thus, results should be carefully interpreted considering symptoms and not using molecular tests alone [86,87,91,92]. Thus, the best approach to optimize the diagnosis of CDI is to combine two tests in an algorithm. The first test should one with a high negative predictive value, including GDH, EIA, or NAAT, and the second test should be toxin A/B EIAs (a test with a high positive predictive value). The GDH/NAAT-based algorithm has reported sensitivity from 91% to 98% and specificity from 96 to 98% [93]. Despite ribotypes producing only CDT are not common in humans, infection with these strains is a challenge at human diagnostic testing, as most tests detect only toxins A and B or its genes. The dissemination of CDT^+^ strains, such as RT027, has caused that some commercial tests also evaluate the presence of CDT genes [94]. Six commercial real-time PCRs (qPCR) that detect CDT are available: The Cepheid Xpert *C. difficile* BT assay (Sunnyvale, CA, USA) [94], the EasyScreen *C. difficile* Reflex (Genetic Signatures, Newtown, NSW, Australia) [95], the VeriGene *Clostridium difficile* Test (Luminex, Austin, TX, USA) [96], the *C. difficile* DNA Complete Test (OpGen, Rockville, MD, USA) [94], the GenSpeed C. diff OneStep Test System (Greiner Bio-One International GmbH, Kremsmünster, Austria) [97] and the GenoType Cdiff (Hain Lifescience GmbH, Nehren, Germany) [94]. In addition, a loop-mediated isothermal amplification-based (LAMP) assay has been described; this assay has the potential to be used as a rapid, reliable and cost-effective tool for detecting CDT^+^ at the point of care [92].

Other approaches have been developed to detect CDT: a method based on MALDI-TOF (matrix-assisted laser-desorption time-of-flight) technology, with the potential to reduce the need for time-consuming molecular methods; and a prototype and research-use only EIA. The latter technique detects CdtB and has a high correlation between detection of fecal CdtB and the recovery of ribotype 027 isolates that produce CDT in vitro [70].

In conclusion, the low prevalence of CDT^+^ strains complicates epidemiological research; however, identification of these strains using systems capable of detecting CDT will help to establish the clinical implications of CDT production and disease.

## 7. CDT as a Therapeutic Target

Antibiotic treatment is the main approach in CDI; however, current treatment with antibiotics such as metronidazole, vancomycin, or fidaxomicin may result in disturbance of the gut microbiota, increasing the risk of recurrent episodes [11]. Toxins A and B have been used as targets for novel therapeutic approaches; nevertheless, owing to the increasing relevance of CDT as a virulence factor and its role in disease, components of CDT are currently used as targets to develop effective new therapeutic strategies for treating hypervirulent strains in particular [91].

Chloroquine and chloroquine derivatives (azolopyridinium salts and 4-aminoquinolines) block CDTb pores in lipid bilayer membranes [98]. These compounds inhibit CDTb-induced Vero cell rounding, supporting the hypothesis that CDTb alone is a pore-forming toxin and suggesting the use of pore blockers as potential therapeutic strategies directed at CDT. Colon epithelial cell lines, HCT 116 and CaCo-2, were also protected against toxin effects by chloroquine derivatives. Moreover, the CDTb-induced loss of epithelial barrier integrity of a CaCo-2 cell monolayer was inhibited by a chloroquine derivative [98].

α-defensin-5 is produced by enteric Paneth cells in the crypts of Lieberkühn to prevent excessive colonization by microorganisms; a neutralizing effect of α-defensin-5 toward TcdA, TcdB, and CDT has been demonstrated [99]. Determination of toxin-induced changes in cell morphology, intracellular substrate modification, and decrease in transepithelial electrical resistance indicated that the inhibition of cell intoxication was time and concentration dependent. For CDT, α-defensin-5 promotes the inactivation of the CDTb pore, which causes marked changes in cell morphology and cell viability. This human peptide may be a candidate pharmacological inhibitor to treat CDI caused by CDT-producing strains [99]. Similarly, α-defensin-1 protects cells and human intestinal organoids from the cytotoxic effects of TcdA, TcdB, CDT, and their combination. In mice, α-defensin-1 reduced the CDT-induced intestinal damage in a time- and concentration-dependent manner. The mechanism of action seems to be based on an interaction between the binding and transport component CDTb and α-defensin-1, leading to direct inhibition of the CDTb channels. It is suggested that α-defensin-1 inhibits oligomer formation of CDTb or blocks the reconstitution of CDTb into lipid bilayer membranes [100]. It was recently shown that CDT binds to Hsp90, Hsp70, and peptidyl-prolyl cis/trans isomerases belonging to the cyclophilin (Cyp) and FK506-binding protein (FKBP) families; furthermore, these proteins are needed for the translocation of components of CDT from endosomes to the cytosol [101,102,103]. Simultaneous inhibition of these chaperones by Rad (inhibitor of Hsp90), CsA (inhibitor of Cyps), FK506 (inhibitor of FKBPs), VER (inhibitor of Hsp70, Hsc70 and Grp78), and Bafilomycin A1 (BafA1, inhibitor of v-ATPase), protects cells from intoxication with CDT; when a combination of inhibitors was used, an enhanced effect was observed [104].

A tetravalent vaccine containing attenuated binary toxin components as well as TcdA and TcdB has been described [105]; it induced the production of neutralizing antibodies against the binary toxin complex in hamsters immunized with either CDTa or CDTb; the combination of CDTa and CDTb had an additive effect on the neutralizing antibody titer. The inclusion of CDTa and CDTb improved the efficacy of the vaccine against NAP1 strains, significantly enhancing survival in hamsters compared with a vaccine containing only attenuated TcdA and TcdB and producing a neutralizing antibody response to TcdA, TcdA, and CDT. Further evaluation of vaccines is required to prevent severe disease by hypervirulent strains of *C. difficile* [105].

## 8. Concluding Remarks

The role of CDT in CDI remains debated; however, evidence suggests an important role of CDT in the pathogenesis of CDI. Several CDT^+^ strains are frequently found and reported and are associated with severe disease (including hypervirulent strains); moreover, strains producing only CDT are becoming prevalent. Thus, the study of these strains is crucial in deciphering the potential contribution of CDT to disease and the changing epidemiology of this emerging pathogen.

## Figures and Tables

**Table 1:** Epidemiology and characteristics of binary toxin-producing strains.

RT	Toxin Genotype	ST	Clade	Characteristics	References
023	*tcdA*^+^, *tcdB*^+^, *cdtA*^+^, *cdtB*^+^	5, 22, 25	3	Resistance to erythromycin, levofloxacin, and moxifloxacin. Reports from USA, Northern and Eastern Europe.	[45,46,47]
027/176	*tcdA*^+^, *tcdB*^+^, *cdtA*^+^, *cdtB*^+^	1	2	Strain associated with increased morbidity and mortality. Reports from Korea, Singapore, Austria, Belgium, Denmark, Finland, France, Germany, Hungary, Ireland, Luxembourg, The Netherlands, Norway, Spain, Sweden, UK, Chile, Panama, Costa Rica, Mexico, Japan, China.	[33,48,49,50,51,52,53,54,55]
033	*tcdA^−^*, *tcdB^−^*, *cdtA*^+^, *cdtB*^+^	ND	5	Isolated from a young patient with ulcerative colitis and severe diarrhea in Australia.	[56,57]
078/126	*tcdA*^+^, *tcdB*^+^, *cdtA*^+^, *cdtB*^+^	11	5	Community-associated and zoonotic strain with increased morbidity and mortality. Reports from France, Italy, Germany, Taiwan, Czech Republic, Korea, Japan, Australia.	[5,40,41,48,50,57,58,59,60,61]
244	*tcdA*^+^, *tcdB*^+^, *cdtA*^+^, *cdtB*^+^	41	2	Community-associated; cause of outbreaks. Reports in Australia, New Zealand.	[44,45,62]
251	*tcdA*^+^, *tcdB*^+^, *cdtA*^+^, *cdtB*^+^	231	2	Isolated from three patients in Australia with severe diarrhea, recurrent disease, and one death.	[63]
826	*tcdA*^+^, *tcdB*^+^, *cdtA*^+^, *cdtB*^+^	ND	5	Identified in an outbreak in The Netherlands, associated with recurrent and severe disease in two of five patients	[64]
ND	*tcdA*^+^, *tcdB*^+^, *cdtA*^+^, *cdtB*^+^	201	3	Isolated from a patient in China, with a severe clinical phenotype; it exhibits a faster germination rate, higher motility, and a higher biofilm formation than RT027 and RT078.	[62,65,66]
ND	*tcdA^−^*, *tcdB^−^*, *cdtA*^+^, *cdtB*^+^	11	5	Isolated from a patient in Germany, with eight episodes of CDI ranging from mild to severe symptoms.	[67]

RT: ribotype; ST: Sequence type; ND: no data; CDI: *Clostridioides difficile* infection.

## Data Availability

The data presented in this study are available in this article.
